# Key indicators for monitoring food system disruptions caused by the COVID-19 pandemic: Insights from Bangladesh towards effective response

**DOI:** 10.1007/s12571-020-01083-2

**Published:** 2020-07-15

**Authors:** T. S. Amjath-Babu, Timothy J. Krupnik, Shakuntala H. Thilsted, Andrew J. McDonald

**Affiliations:** 1 International Maize and Wheat Improvement Center (CIMMYT), Dhaka, Bangladesh; 2 WorldFish, Penang, Malaysia; 3 School of Integrative Plant Science, Cornell University, Ithaca, NY USA

**Keywords:** COVID-19, Food system disruption, South Asia, Monitoring, Resilience

## Abstract

In the context of developing countries, early evidence suggests that the impacts of the COVID-19 pandemic on food production systems is complex, heterogenous, and dynamic. As such, robust monitoring of the impact of the health crisis and containment measures across agricultural value chains will likely prove vitally important. With Bangladesh as a case study, we discuss the building blocks of a comprehensive monitoring system for prioritizing and designing interventions that respond to food system disruptions from COVID-19 and preemptively avoid further cascading negative effects. We also highlight the need for parallel research that identifies pathways for enhancing information flow, analysis, and action to improve the efficiency and reliability of input and output value chains. In aggregate, this preliminary work highlights the building blocks of resilient food systems to external shocks such as COVID-19 pandemic in the context of developing nations. In doing so, we call attention to the importance of ‘infection safe’ agricultural input and output distribution logistics, extended social safety nets, adequate credit facilities, and innovative labor management tools alongside, appropriate farm mechanization. In addition, digital extension services, circular nutrient flows, enhanced storage facilities, as well as innovative and robust marketing mechanisms are required. These should be considered in parallel with effective international trade management policies and institutions as crucial supportive measures.

## Introduction

The COVID-19 crisis has affected the world in an unprecedented way (Loayza and Pennings 2020). In addition to the public health effects of the disease, measures to contain the spread of COVID-19 pose significant risks to food and nutrition security through disruptions to food production, distribution, and access. Few previous shocks have had such a significant range of effects on food systems in such a short period (CCAFS 2020). The World Health Organization (2020) reported the first COVID-19 cluster of cases on December 31, 2019. Since then, researchers have grappled to understand the complexity of the pandemic on food and nutrition – especially in developing nations (Galanakis 2020). In this viewpoint, we use Bangladesh as a case study region to propose indicators to systematically monitor the health of food production systems in the developing country context. Following discussion of the monitoring system, response mechanisms for rapid recovery from shocks and potential mechanisms to pre-emptively avoid further cascading negative effects are proposed.

## COVID-19 food production system disruption pathways

**Fig. 1:**
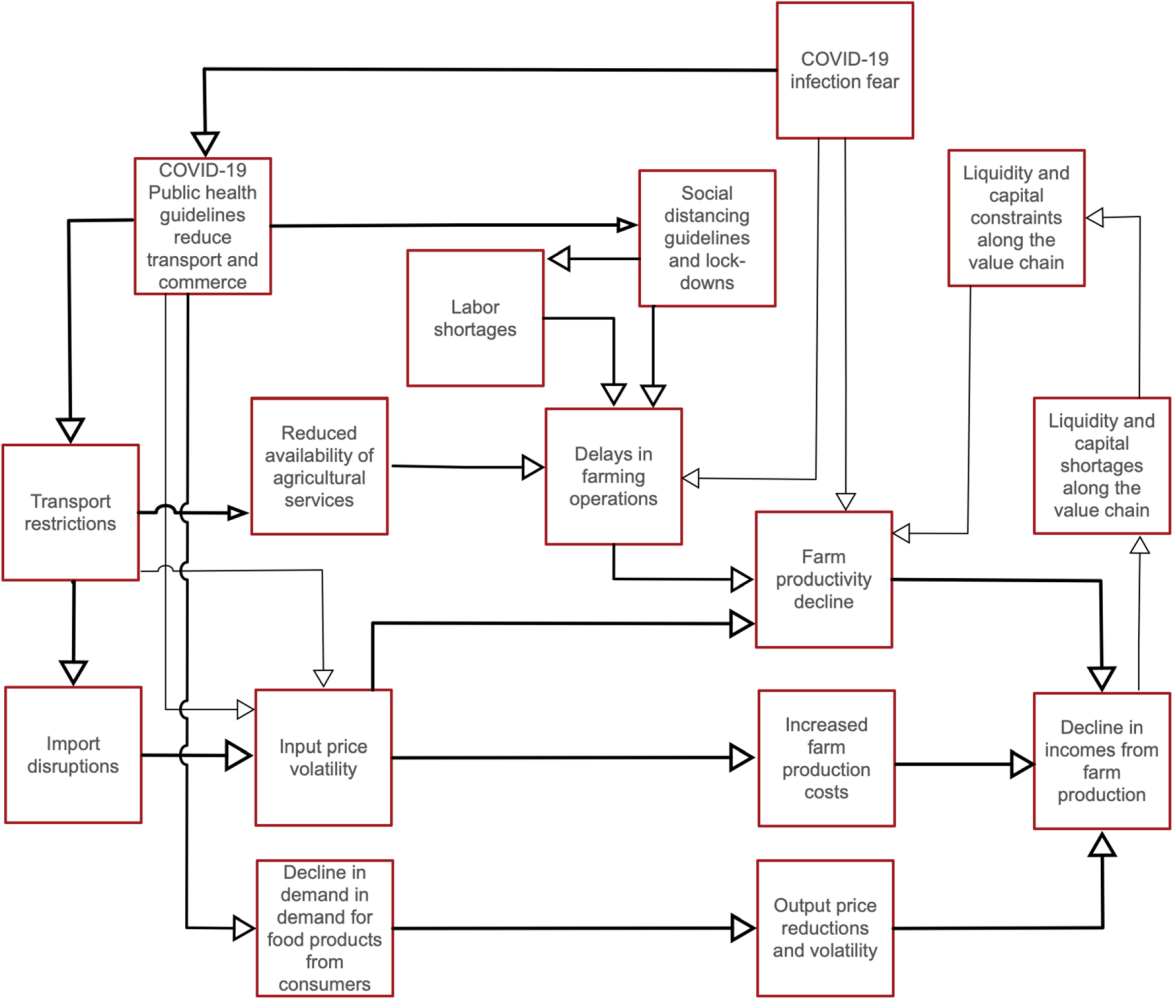
Flow chart depicting potential COVID-19 impact pathways that may affect food production systems in South Asia. (Note: thickness of arrows shows strong relation as assessed by experts)

Our synthesis of media reports and rapid field assessments suggests that South Asia’s food production systems – and particularly those reliant on external inputs and human labor – are being disrupted by the COVID-19 crisis through multiple pathways. International and domestic supply chains for fertilizers, agro-chemicals, machinery, and seeds have been impeded by import and movement restrictions. Logistical constraints (international shipping and domestic transport interruptions), and partial closures of input dealerships appear to be common problems. Similar disruptions to hatchery operations and feed supplies (FAO 2020a) affect aquaculture, poultry, and livestock production. ‘Stay in place’ orders and limitations imposed on migration have created pockets of labor shortages (FAO 2020b), particularly for the harvest of South Asia’s winter *‘rabi’* season crops including rice, wheat, and horticultural crops (ICRISAT 2020). Even where farmers have been able to hire sufficient farm labor, social distancing measures have slowed operations. Where farming systems are more mechanized, transport restrictions have inhibited the movement of agricultural machinery, although efforts taken by regional governments to permit unfettered movement of agricultural equipment and goods has resulted in some degree of success (USAID 2020; FAO 2020c). Concerns have emerged that there could be planting delays – mainly for rice – in the subsequent *‘kharif*’ (monsoon) season. In addition to labor, shortages in seed availability may also complicate planting. In India, farmers across 43 districts have indicated insufficient seed availability for the *kharif* crop (PRADAN 2020). Crop establishment delays in the monsoon season can have ‘knock-on’ effects (exposure to late season terminal heat stress) on the following *‘rabi*’ (winter) season crop by delaying its establishment (Dubey et al. 2020; Arshad et al. 2017). Similarly, aquaculture and livestock activites have also faced delays in stocking, feeding, and other operations, reducing potential production and complicating timely marketing. Disruption of hatchery operations and processing facilities due to logistic, labour and input related constraints have also become concerns (FAO 2020c). In addition to production disruptions, farmers also face output market challenges, with fewer buyers willing to purchase products, particularly for perishables. Commodities with high income elasticities such as fruits and vegetables, meat, fish, milk, and eggs are facing significant declines in demand due to contraction in incomes of non-salaried informal workers and price spikes, especially in urban areas (Abhishek et al. 2020). In addition, closures of restaurants and food catering businesses have also affected demand, especially of fish and livestock products (FAO 2020a). For individual farmers, lower sales revenues that result from price and sales volume changes for winter season produce have led to capital shortages. These in turn could impact input purchasing decisions, especially for the 2020 monsoon *kharif* cropping season that commenses in July. The aggregate economic impacts of these developments are likely to be transferred along agricultural value chains (Saghaian et al. 2008; Hassouneh et al. 2012). In addition, a reduction in sales of agricultural inputs is likely to translate into reduced cash flows for retailers and wholesalers, which may lead to a liquidity crunch that can impact dealers’ ability to maintain stocks of critical inputs (iDE 2020). In sum, these interactive effects could cascade throughout the food system (Fig. 1).

## Monitoring the health of food production systems

Because food production systems are complex, the assessment of COVID-19’s impact on their functioning and ‘health’ requires monitoring and data at each step of the value chain – from inputs to processing and distribution, and to sales. To our knowledge, there are relatively few examples of efforts to regularly ‘take the pulse’ of the functioning of food production systems from a value chain perspective, and the shock caused by the COVID-19 crisis has underscored the importance of regular diagnostics along the value chain using key indicators. Monitoring is also crucial for efforts to accelerate recovery and ensure resilience of the food system (Peterson et al. 2018). Table 1 describes some of the major constituents of a comprehensive monitoring regime to permit a systematic assessment of food production systems. Such monitoring will not only signal problems, but also help guide the design of interventions aimed at faster recovery, and ultimately to improve food systems resilience.

**Table 1:** Monitoring indicators for food production systems performance disruption

Productivity factors		Data types and sources
Internal farm factors	Labor	• Spatially-explicit family labor opportunity costs and hired labor costs. Studies of hired labor availability and scarcity.
	Resource supply and management factors	• Spatially-explicit farmer-survey based diagnostic studies of farmers’ ability to supplement external inputs of nutrients with regenerative agricultural techniques, (e.g., productive options for low-external input agriculture, with coupling to pest management techniques) • Comprehensive studies of access to water and irrigation resources at the farm level • Inventories of farm machinery and equipment • Inventories of animal, fish, and poultry stock and productivity levels
Internal or external	Seed	• Seed replacement rates and seed availability studies for key varieties at the farmer level • Seed stock reserves available with public and private agencies and seed demand forecasts. Seed production information from key hybrid seed suppliers
External inputs	Fertilizers	• Current fertilizer stock and price information, and sufficiency assessment at the manufacturer, distributor, retailer levels, coupled with production forecasts at the manufacturer level • Fertilizer import data and fertilizer demand estimates • Global fertilizer production and shipment data from international agencies, e.g., from the International Fertilizer Association
	Pest control products	• Availability and prices of key pesticides, veterinary and aquacultural medicines sourced from surveys at the farmer, input retailer, manufacturer, and import-broker levels • Import country mapping of the supply chain for widely used products to facilitate an understanding of possible disruptions (if countries are dependent on imports for particular products) • Feasibility studies for alternative products (e.g., locally available biopesticides or biocontrol agents if pest threats are severe and availability of the chemicals are limited)
	Livestock and aquaculture	• Studies at the input dealer and industry level on the availability and costs of animal feed, fingerlings, brood stock, chicks, calves and heifers • Information on production figures from hatcheries compared to normal volumes
Agricultural services	Farm machinery	• Readiness of machinery factories in shipping new and anticipated orders for machines and parts • Cost and service type data from agricultural machinery service providers, dealers, manufacturers, and importers indicating the availability and prices for four-wheel and two-wheel tractors, transplanters, seed drills, irrigation pumps, harvesters and reapers, and post-harvest equipment • Diagnostic surveys providing time-series data on the prevailing cost and payment modality for services from these machineries to farmer-clients • In the case of irrigation and harvesting services, remotely sensed data on areas with localized water deficits and area of mature and harvested crop fields (to target services delivery)
	Extension	• Comprehensive databases of governmental, NGO and private sector extension agents with contact information including telephone number, email, social media identification • Inventory of extension agent specializations
	Logistics	• Panel data on cost and vehicle availably data from courier services, trucking and shipping companies • Agricultural goods and equipment import and export records, including the rate at which imports clear for distribution after arrival in ports
	Credit and capital	• Credit disbursal data from commercial and agricultural banks, micro finance institutions, and informal banking sources • Farm level data related to cash liquidity and credit availability for purchasing inputs
Processing and marketing	Processing and mills	• Time series data on the volume, costs of processing, and sales of processed goods • Studies to assess the degree to which imports of processed goods and feed can be substituted • Data on milk purchases by processing companies and prices
	Output market	• Data from wholesale traders and middlemen, data from feed and grain mills • Monthly international price data published by USDA and/or FAO, in addition to other sources • Consumer level price data on cereals, vegetables, milk, meat, fish and poultry

## Portfolio of potential response mechanisms

To provide insight into a potential portfolio of response mechanisms against the disruption in food production systems caused by the COVID-19 pandemic, we use Bangladesh - a country of 164.7 million people with considerable food and nutrition security concerns (WFP 2016) - as a case study. Initial estimates show that COVID-19 impacts have pushed an additional 20% of people below poverty line in Bangladesh (PPRC-BIGD 2020). In April of 2020, four CGIAR institutions working in Bangladesh (CIMMYT, IFPRI, IRRI and WorldFish) submitted a formal letter to the Secretary of the Ministry of Agriculture outlining the scale of the COVID-19 impact on food systems, while also presenting potential avenues for corrective actions (CSISA 2020). The potential solutions presented here are based on the suggestions presented in in this letter.

### Crucial national actions

#### Expanding social safety nets for the extreme poor

First and foremost, there is an immediate need to strengthen social safety nets programs that can assist in assuring basic income and food access (Gilligan 2020). This includes support to smallholder and resource-poor farmers and producers in rural areas, but also to urban poor and rural landless consumers who are experiencing elevated prices for food products (NAWG 2020, PPRC-BIGD 2020). The government of Bangladesh has expanded social safety net programmes, and possibilities exist to further modify existing models to enhance demand for agricultural products, a step that will aid both food producers and consumers. The safety net schemes that provide cash in exchange for labor can be modified to a *food and cash for work* programme that could be linked to initiatives to increase demand for domestically and locally-sourced farm produce while protecting food security for the poor. Here, manual work for a willing section of the population could be compensated in part by cash and also by food (fresh produce purchased from farmers by government and resupplied to workers), potentially leading to synergies in development and recovery objectives (increased farm income and food security). This can lead to triple wins if manual labour can be used to create durable ‘public goods’ such as food storage or market facilities, or infrastructure improving climatic resilience (e.g. shelters from extreme weather events, water harvesting and storage, or flood protection structures) that can enhance agricultural productivity and societal resilience the longer-term. Given the current slump in demand for income elastic farm commodities, food for work safety net interventions need to include a diverse range of products including vegetables, fruits, milk, and eggs to protect farm incomes and enhance nutritional security.

This model could, however, be more complex to operate than cash for work programmes due to the logistics involved. Measures to stimulate demand (e.g., mobile farm goods markets using open-air flatbed trucks that reduce the potential for crowding, COVID-19 safe ‘contactless’ home delivery systems in urban areas) and dispel myths around the consumption of animal-sourced foods and disease infection (Islam et al. 2020) could aid in recovery while stimulating markets. Modified market handling procedures and COVID-19 safe farm market operation guidelines (e.g., protocols for safe handling and cleaning of agricultural machinery, storage infrastructure, transport equipment, and sanitary wet-markets) may be of help, especially in urban areas. Given the current urban population exceeding 60 million in Bangladesh and growing at 3% per year, these measures could help in revitalizing market demand (World Bank 2020). Campaigns to develop science-based perceptions of food safety will also likely be important.

#### Maintaining the flow of key inputs and outputs

Secondly, in systems where production depends on external inputs, market systems must be maintained. In Bangladesh and other countries in South Asia, agricultural inputs and produce are widely transported through informal courier services, on animal carts, and uncovered vehicles (FAO 2009). To our knowledge, none of the countries of South Asia including Bangladesh have made sufficiently clear exemptions during lock-down periods for these diverse forms of transportation to move agricultural goods. Such exemptions would also require assurances for improved safety, ideally through formal protocols to reduce risks of disease spread. These suggestions also require supporting the private sector in its crucial role to provide affordable inputs to farmers when and where they are needed. Supportive measures can include exemptions for permitted working hours for input retailors or creating alternative contactless models of farm input deliveries that ensure social distancing and prevent disease spread. Similarly, the flow of food products from rural to urban areas needs to be facilitated. Guaranteeing the supply of horticultural, fish and livestock products – in addition to staples such as rice and wheat – is required to ensure diverse, nutritious and safe diets.

#### Minimizing disruption in agricultural services

Farmers in South Asia and Bangladesh are widely dependent on rural migratory laborers or agricultural machinery owners who offer land preparation, planting, irrigation, harvesting and post-harvesting services to farmers on a fee-for-service basis (Mottaleb et al. 2016). Many of these operations are crucial for agricultural productivity. Social distancing measures and the shortage of manual labor and machinery services have already been shown to disrupt harvesting (FAO 2020c, NAWG 2020). Governmental support and potential cost-offsets for the provision of scale-appropriate farm machinery transportation, machine purchases, and services may provide substantial benefits in this period. As an example, the government of Bangladesh recently allocated 0.37 billion USD to support appropriate farm mechanization as a part of COVID-19 mitigation response (Dhaka Tribune 2020). Creation of labour banks (a pool of willing and healthy workers who can be readily contacted, assembled, and deployed) can also act as an intermediary measure to tackle labour shortages, so long as proper operating protocols for social distancing and other diesease preventative measures are understood and enforced.

Increasing storage for perishables through increased cold and hermetic storage represents an additional action that could help to avoid wastage of agricultural products. To address reductions in farmers’ ability to access extension services, strengthening digital and telephonic extension services could prove beneficial. Examples include use of tele-networks and interactive voice message services (CIMMYT 2019), early warning and agricultural advisory systems (eg: https://www.agvisely.com/), and smartphone apps (Faruq 2017). Many of these tools are already available and can be strengthened in Bangladesh and South Asia.

#### Ensuring access to financial services

Ensuring financial liquidity and the purchasing power of farmers is crucial for maintaining healthy food production systems. For capital-intensive systems like aquaculture, credit availability largely determines productivity (Mitra et al. 2019). Revenue declines due to lower sale prices, reduced production, or harvest losses, or difficulties accessing output markets, can erode liquidity and constrain capital availability for the next cycle of farming operations. Temporary measures like diverting productive capital for consumption purposes and reduced flow of remittances to rural areas can increase capital shortages (Gurenko and Mahul 2004) This can in turn increase demand for credit. Expanding access to affordable finance options (like low interest credit with less stringent terms) including digital finance services for farmers may prove useful to ensure sufficient use of critical inputs to stabilize production - both during and after the pandemic.

### Maintaining the flow of international trade

In addition to domestic actions, internationally oriented interventions are also crucial. For example, 60% of the urea and more than 90% of the Muriate of Potash (MOP), Triple Super Phosphate (TSP), Diammonium Phosphate (DAP) fertilizers used by farmers in Bangladesh are imported (BFA 2020). Bangladesh is also dependent on the regular import of commodities like wheat (6 million tonnes year^–1^), soy (1 million tonnes year^–1^), and maize (2 million tonnes year^–1^) for both human food and animal feed (USDA 2019). Hybrid seeds (e.g. for maize and vegetables) are also commonly imported. This indicates the vulnerability of food production and consumption to international supply disruptions, partially underscoring the need for domestically robust seed supply and circular economy interventions that prioritize nutrient recycling. That said, transition to more circular and self-reliant systems will not happen quickly and is likely to be only a partial solution. More importantly, the current crisis requires steps that ensure resilience and diversification in international supply chains. Efforts to prioritize the continued functioning of port operations to facilitate the flow of agricultural trade will be required. In Bangladesh, even partial closure of ports may result in high prices and limited stocks of grains, pulses, edible oils as well as crucial feed supplies (particularly maize and soybean). Similarly, although current national input stocks appear to be sufficient for the near future, prolonged suspension of international trade could undermine the post monsoon season supply of key inputs (particularly fertilizers, vaccinations, medicines used in aquaculture, hybrid seeds and potentially fuel for machinery and irrigation equipment) at reasonable prices.

These factors also highlight the need for additional research that investigates methods to build value chain resilience of farm, fish, and livestock systems. Alternate import arrangements, circular flow of nutrients from urban bio- and food-wastes (Therond et al. 2017; Sengupta et al. 2015), and a renewed focus on input resource use efficiency (Darnhofer 2020) could provide a buffer from external shocks. To address risks to the continued use of appropriate agricultural machinery, emphasis should be given on the development of short-term (e.g., potential use of 3D printers to assist in fabrication of spare parts for agricultural machinery) alongside medium- to long-term solutions (e.g., formal mass production of spare parts followed by industrial development of the domestic light engineering sector).

## Conclusions

Well-designed monitoring systems can assist in the development of early warning systems to alert when food production systems and associated agricultural value chains are nearing vulnerability thresholds. Monitoring of indicators can assist in facilitating proactive engagement, while also providing a datastream to inform corrective interventions for speedy recovery. This viewpoint provides the outlines of a monitoring and early warning system for food system disruptions in Bangladesh, with ramifications for other developing nations. A COVID-19 resilient food system to is likely to be one that has stable supply chains, infection safe logistics, extended social safety nets, adequate credit facilities, and innovative labour management tools, alongside appropriate farm mechanization efforts. In addition, COVID-19 safe farm operation protocols, digital extension services, circular nutrient flows, enhanced storage facilities, and innovative marketing mechanisms are needed, along with effective international trade management policies and institutions. Given that the actions required to implement monitoring of food systems health and undertake corrective measures are complex and interconnected, creation of an adequately funded institutional mechanism to coordinate monitoring and mitigation measures could be beneficial. The anticipated longer-term nature of the COVID-19 crisis – which is still unfolding in South Asia and globally – is another compelling reason. In addition, increased coordination with different government, private, and non-governmental agencies, as well as development partners, multilateral institutions, and international agencies are needed for successful mitigation and the creation of more resilient food systems.
